# Supplementary material for: CD14 Expressing Precursors Give Rise to Highly Functional Conventional Dendritic Cells for Use as Dendritic Cell Vaccine
Source: Cancers (Basel). 2021 Jul 29;13(15):3818. doi: 10.3390/cancers13153818 (PMC8345076; doi:10.3390/cancers13153818)
Supplement: Supplementary file 1 [file cancers-13-03818-s001.zip › Supplementary figures_V2.pdf]

Supplementary Figure S1

cl 0

GO:0016071	mRNA metabolic process
GO:0044265	cellular macromolecule catabolic process
GO:0008380	RNA splicing
GO:0006397	mRNA processing
GO:0034622	cellular protein-containing complex assembly

cl1

GO:0000278	mitotic cell cycle
GO:0140014	mitotic nuclear division
GO:1903047	mitotic cell cycle process
GO:0007049	cell cycle
GO:0000280	nuclear division

cl 2

GO:0002444	myeloid leukocyte mediated immunity
GO:0002274	myeloid leukocyte activation
GO:0016050	vesicle organization
GO:0043299	leukocyte degranulation
GO:0002275	myeloid cell activation involved in immune response

cl 3

GO:0001775	cell activation
GO:0002274	myeloid leukocyte activation
GO:0043299	leukocyte degranulation
GO:0045321	leukocyte activation
GO:0002444	myeloid leukocyte mediated immunity

cl 4

GO:0016032	viral process
GO:0044403	symbiotic process
GO:0044419	interspecies interaction between organisms
GO:0002252	immune effector process
GO:0016050	vesicle organization

cl 5

GO:0006614	SRP-dependent cotranslational protein targeting to membrane
GO:0006613	cotranslational protein targeting to membrane
GO:0000184	nuclear-transcribed mRNA catabolic process, nonsense-
GO:0045047	protein targeting to ER
GO:0019080	viral gene expression

cl 6

GO:0002274	myeloid leukocyte activation
GO:0001775	cell activation
GO:0002252	immune effector process
GO:0002444	myeloid leukocyte mediated immunity
GO:0043299	leukocyte degranulation

cl 7

GO:0006413	translational initiation
GO:0006414	translational elongation
GO:0006412	translation
GO:0043043	peptide biosynthetic process
GO:0000184	nuclear-transcribed mRNA catabolic process, nonsense-

cl 8

GO:0042254	ribosome biogenesis
GO:0006364	rRNA processing
GO:0016072	rRNA metabolic process
GO:0022613	ribonucleoprotein complex biogenesis
GO:0034660	ncRNA metabolic process

Supplementary Figure S1. Top 5 Gene ontology terms obtained from the differentially expressed genes (DEGs) of the clusters obtained from single cell RNA sequence data. The GO terms of candidate DEGs were analyzed using ToppGene.

Supplementary Figure S2

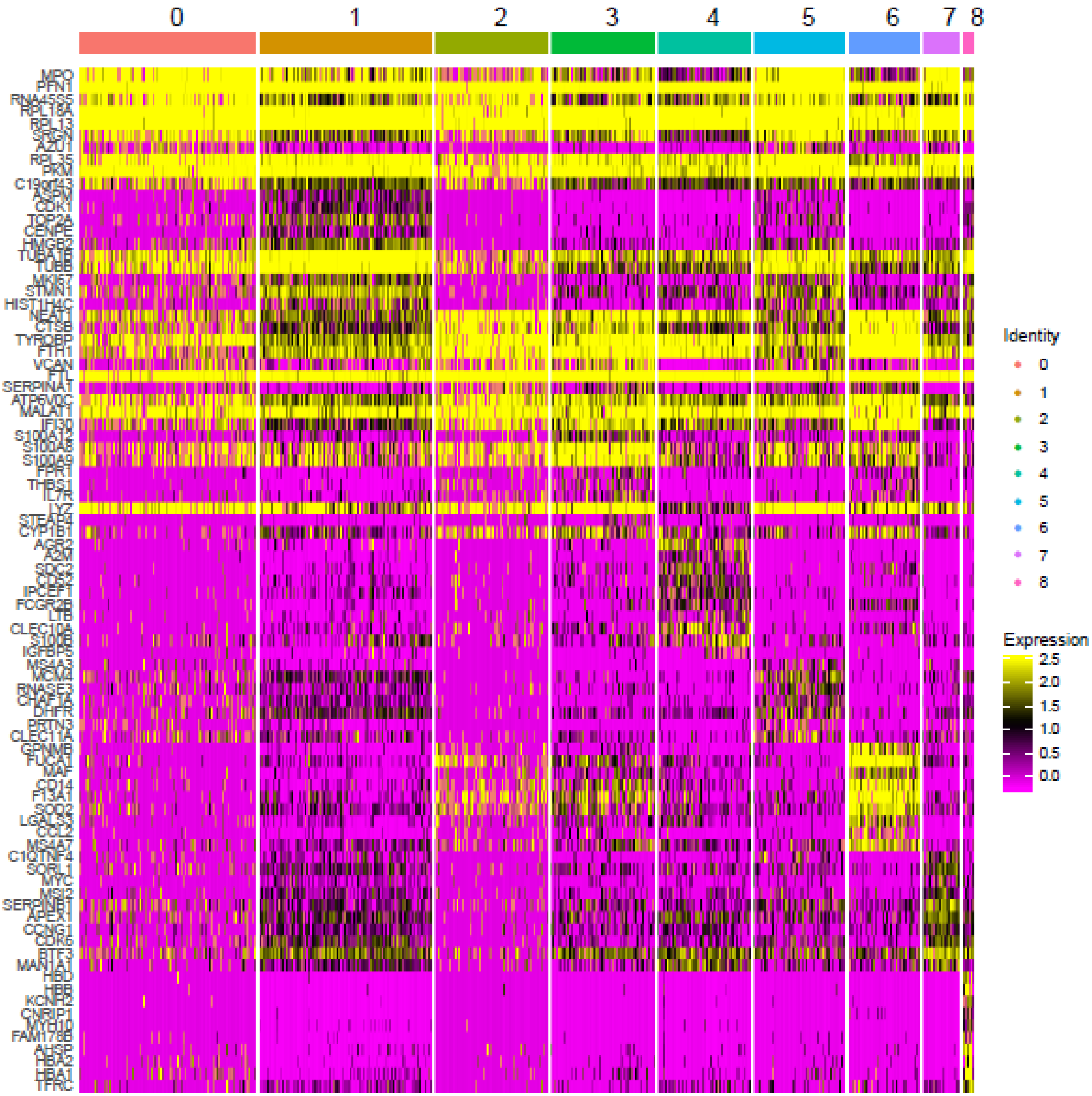

Supplementary Figure S2. Heatmap of the top 10 differentially expressed genes (DEGs) of the clusters obtained from single cell RNA sequence data.

Supplementary Figure S3

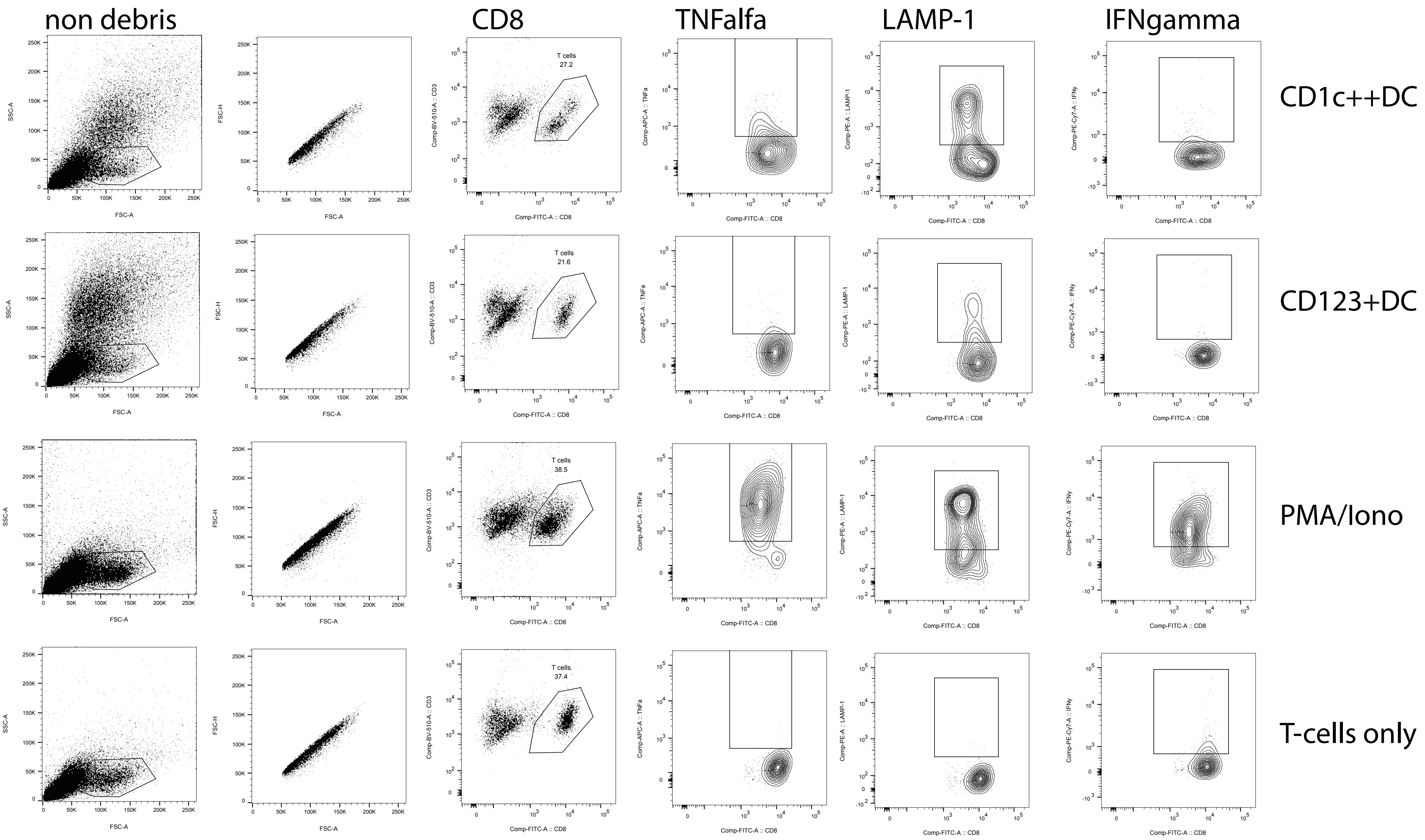

Supplementary Figure S3. Gating strategy for WT1-specific T-cell assay. One experiment is shown out of 5. Live lymphocytes are gated followed by CD8 and accordingly TNFalfa, LAMP-1 or IFNgamma.
